# Misokainia

**Published:** 1907-07

**Authors:** 


### "MISOKAINIA."

Achilles Rose has invented the term "misokainia" to designate that disease which is characterized by a tendency to combat new ideas and discoveries. As is well said by Morgenstern, this is one of the great bars to human progress.

Since this establishes the affection in question as a disease, that is, an abnormal, morbid condition, it behooves us to be on the lookout for it. Unfortunately it is especially prevalent among physicians. The effects are disastrous, consisting largely of a sclerosis and congelation affecting the cerebral tissues and the heart. The earliest symptoms are an indisposition to receive and assimilate new ideas. This is soon followed by an unwillingness to acknowledge that the victim is capable of receiving new ideas of value. The conviction is apparent in his mind that his professional knowledge is so completely rounded out and perfected that an addition thereto is an impossibility.—American Jour. Clin. Med.

